# Improved quantitation and reproducibility in multi-PET/CT lung studies by combining CT information

**DOI:** 10.1186/s40658-018-0212-0

**Published:** 2018-06-05

**Authors:** Beverley F. Holman, Vesna Cuplov, Lynn Millner, Raymond Endozo, Toby M. Maher, Ashley M. Groves, Brian F. Hutton, Kris Thielemans

**Affiliations:** 1 Institute of Nuclear Medicine, University College London, UCLH (T-5), Euston Road, London, NW1 2BU UK; 2 National Institute for Health Research Respiratory Biomedical Research Unit, Royal Brompton Hospital, Sydney St, London, SW3 6NP UK; 3 Fibrosis Research Group, Inflammation, Repair and Development Section, NHLI, Sir Alexander Flemming Building, Imperial College London, London, SW7 2AZ UK; 4 Centre for Medical Radiation Physics, University of Wollongong, Wollongong, Australia

**Keywords:** Lung, Density, PET/CT, Respiration, Attenuation correction, Quantitation, Dynamic PET

## Abstract

**Background:**

Matched attenuation maps are vital for obtaining accurate and reproducible kinetic and static parameter estimates from PET data. With increased interest in PET/CT imaging of diffuse lung diseases for assessing disease progression and treatment effectiveness, understanding the extent of the effect of respiratory motion and establishing methods for correction are becoming more important. In a previous study, we have shown that using the wrong attenuation map leads to large errors due to density mismatches in the lung, especially in dynamic PET scans. Here, we extend this work to the case where the study is sub-divided into several scans, e.g. for patient comfort, each with its own CT (cine-CT and ‘snap shot’ CT). A method to combine multi-CT information into a *combined-CT* has then been developed, which averages the CT information from each study section to produce composite CT images with the lung density more representative of that in the PET data. This combined-CT was applied to nine patients with idiopathic pulmonary fibrosis, imaged with dynamic ^18^F-FDG PET/CT to determine the improvement in the precision of the parameter estimates.

**Results:**

Using XCAT simulations, errors in the influx rate constant were found to be as high as 60% in multi-PET/CT studies. Analysis of patient data identified displacements between study sections in the time activity curves, which led to an average standard error in the estimates of the influx rate constant of 53% with conventional methods. This reduced to within 5% after use of combined-CTs for attenuation correction of the study sections.

**Conclusions:**

Use of combined-CTs to reconstruct the sections of a multi-PET/CT study, as opposed to using the individually acquired CTs at each study stage, produces more precise parameter estimates and may improve discrimination between diseased and normal lung.

## Background

PET/CT is becoming more popular for use in diffuse lung diseases such as chronic obstructive pulmonary disease (COPD), interstitial lung disease (ILD), infection and inflammation [1]. These studies commonly address disease progression and treatment effectiveness and make use of quantitative dynamic as well as static imaging [2–6].

Quantitative PET images can only be obtained with accurate attenuation correction (AC) maps. These AC maps are commonly derived from short CT acquisitions (i.e. a standard helical CT for AC), which are appropriate in regions where internal anatomical motion is unlikely [7, 8]. However, in regions such as the thorax, these CT techniques only produce a ‘snap shot’ of the respiratory cycle and therefore determining an AC map matched to the PET data is difficult [9].

Respiratory AC map mismatches are due to two main contributions; motion, which leads to anatomical location mismatch, and density variations due to lung fractional air volume changes over the course of the breathing cycle [10], which could lead to attenuation coefficient mismatch. Mismatches due to motion have been widely explored in the literature [11, 12] and although density variations due to the respiratory phase at the time of acquisition have long been acknowledged by the CT community [13, 14], the effect on PET quantitation in the lung has only very recently been acknowledged [15].

In a previous publication [15], the effect of density variations between PET and CT on PET quantitation was explored. In this paper, we expand this work to multi-PET/CT (MPC) protocols. We define MPC protocols as those in which a dynamic PET/CT study is broken into two or more sections, allowing the patients to have short breaks for their comfort so as to improve patient compliance. Each section consists of a CT and a PET acquisition. The aim of the individual CTs is to allow for attenuation correction of the corresponding PET data, while enabling accurate CT-based registration between the study sections. However, the CTs at the start of each study section may capture different phases of the breathing cycle, leading to variations in the degree of mismatch and resulting PET quantitation at each section. In turn, this will lead to errors in the extracted kinetic parameters.

This section-specific mismatch is likely to be most problematic if ‘snap-shot’ CTs are used. If an average of the cine-CTs can be used for attenuation correction [16] for all study sections, it is expected that these will better match the PET acquisitions, but differences can still occur due to changing breathing patterns. As a result, variation in quantification bias across the study sections is not necessarily rectified by using the higher dose cine-CT acquisitions.

In this paper, the errors in estimating kinetic parameters from ungated dynamic non time-of-flight (non-TOF) PET data associated with MPC protocols using ‘snap shot’ CTs are assessed using XCAT phantom simulations. A method is then suggested to reduce the errors in MPC studies (based on either ‘snap shot’ or averaged cine-CT) by combining all the available CT information. This combined-CT method is then tested on non-TOF ^18^F-FDG data from patients who have undergone MPC studies with a mixture of cine-CT and ‘snap shot’ CTs, to determine the improvement in the PET quantitation.

## Methods

### XCAT simulations

Initially, the possible errors in PET quantification as a result of using MPC protocols were investigated. A dynamic study with known kinetics was simulated using the XCAT phantom [17]. The time frames of this dynamic study were broken into three sections to match the MPC imaging protocol timings shown in Table 1 which describes the patient protocol used to acquire dynamic ^18^F-FDG PET/CT of the thorax (see the “Patient acquisitions” section). In the simulation, all three CT acquisitions were assumed to be ‘snap shot’ CTs. Each section was then reconstructed with AC maps corresponding to different stages of the respiratory cycle as outlined below.

**Table 1:** Patient acquisition protocol

Time post injection (min)	Acquisition
	Early section
	Cine-CT (1 PET bed position)
	120 kV, 10 mA, 0.5 s full rotation, 0.2 s cine interval
	Cine duration dependent on respiratory rate
0	Patient injection
0	20-min dynamic PET (1 PET bed position)
	6 × 10 s, 3 × 20 s, 3 × 60 s, 5 × 120 s, 1 ×300 s
20	Patient break (free to move)
	Mid section
32	Cine-CT (1 PET bed position)
	Cine parameters and duration same as in previous acquisition
34	20-min dynamic PET (1 PET bed position)
	4 × 300 s
54	Patient break (free to move)
	Late section
64	CT acquisition for AC whole Lung (2 PET bed positions)
	120 kV, 10 mA, 0.8 s full rotation, helical, pitch 1.375:1
66	Static PET (2 PET bed positions, 3 min per bed)

An ungated PET XCAT simulation, which contains AC map mismatches due to both motion and density (MD), was created as previously described [15] and outlined in brief here. First, tissue time activity curves (TTACs) and associated kinetic parameters (true parameters) for every organ in the thorax were determined from a patient dataset. These were used to create an organ-based activity distribution corresponding to each time frame in the dynamic sequence. For each time frame, XCAT phantoms of the thorax were created for 11 short time periods evenly spaced over a single breathing cycle (equivalent to respiratory phase gated periods), where the breathing motion was defined by the XCAT default diaphragm and anterior-posterior motion curves. An average of these 11 gates was used to form a motion averaged PET image. AC maps for the 11 gates were also simulated varying the density in the lung regions from end expiration (*μ*=0.0231 cm^−1^) to end inspiration (*μ*=0.0194 cm^−1^) (representing a healthy patient). From these gates, an averaged PET-matched AC map was created by averaging all 11 gates (AVE). In addition, mismatched ‘snap shot’ AC maps from the extreme gates representing end inspiration (INS) and end expiration (EXP) as well as a mid-breathing cycle (MID) gates were obtained. PET sinograms were created by using the analytic simulation capabilities of the ‘Software for Tomographic Image Reconstruction’ (STIR) [18] package, based on a model of the GE Discovery VCT scanner. PET volumes for each dynamic frame were forward projected taking into account single scatter and attenuation using the matched AC maps. These PET data were then reconstructed in STIR using OSEM (7 subsets, 40 iterations) with both the correct and mismatched AC maps for attenuation and scatter correction.

To analyse the reconstructed PET data, lung and left ventricle masks (that have been eroded to reduce the effects of motion) were used to obtain time activity curves (TACs) from each of the PET datasets. Following the protocol outlined in Table 1, the derived lung TTACs were separated into early, mid and late study sections, each with three sets of frames derived from the different AC maps. All possible combinations were selected to create 27 full TTACs. An image-derived input function (IDIF) was determined from the left ventricle TAC by fitting a previously defined model [19]. An irreversible two-tissue compartment model [20] was then fit to each of the TTACs, and the influx rate constant *K*_*i*2_ was determined (where the notation 2 is used to represent the influx rate constant determined from the two-tissue compartment model) [21]. Patlak-Rutland analysis was also performed on the TTACs [22, 23] to determine *K*_*iP*_ (where the *P* represents the influx rate constant from Patlak-Rutland analysis). For each TTAC, the percentage difference between the true and measured *K*_*i*_ was determined.

### Patient acquisitions

Nine patients, 5 males and 4 females aged 71 ± 6 years, all with diagnosed idiopathic pulmonary fibrosis (IPF), underwent ^18^F-FDG dynamic PET/CT with the protocol outlined in Table 1 using a GE Discovery VCT PET/CT scanner. All patients were imaged supine immediately post-injection using 209 ± 23 MBq ^18^F-FDG. The study was split into three stages (early, mid and late) to relieve patient discomfort during the long acquisition. The duration of the cine-CTs acquired for early and mid-cycle stages was set to the duration of the patient’s complete breathing cycle plus one extra second. A cine-CT obtains multiple CT acquisitions over time for each patient slice building up a 4D CT dataset [24]. The final stage of the study was a normal clinical static PET with a shallow breathing ‘snap shot’ CT study.

The patient data used in this study were the baseline scans (i.e. prior to patient dosing) obtained from a dose escalation study (NCT01725139) of omnipalisib (GSK2126458) in patients with IPF that included FDG-PET as one of the pharmacodynamics endpoints. Institutional Review Board permission, the UK Medicines and Healthcare Products Regulatory Agency (MHRA) approval and informed patient consent were obtained for the study.

### Patient data: multi-PET/CT method and analysis

In an initial study, the data from the nine imaged IPF patients (the “Patient acquisitions” section) were reconstructed without consideration of the effects of density variations in order to be consistent with the current methods used in the clinic. Each stage of the PET study was individually reconstructed using OSEM (7 subsets, 40 iterations, 6-mm FWHM Gaussian filter) with the corresponding CT for AC (either static CT or averaged cine-CT). GE proprietary software for off-line data processing of PET/CT data was used. The averaged cine-CT from the early and mid study sections were registered using non-rigid registration into the space of the late study (target image). Registration was achieved using the NiftyReg software [25, 26], and the deformation fields from these registrations were used to register all the PET frames. Note that the non-rigid registration using the Niftyreg software only accounts for the repositioning of the patient between study sections; however, density changes are not corrected. Therefore, the deformed images will match in terms of anatomical location, but the original lung density will be unchanged.

An IDIF was determined by manually drawing a 2-cm diameter spherical region of interest (ROI) within the ascending aorta [27] on the registered images. The size of this ROI was chosen to be smaller than the diameter of the ascending aorta in order to minimise partial volume effects [28]. For each patient, the TAC for a selected aorta region was fit using a previously defined model [29] to create the IDIF.

The density in two regions of the lung on each of the registered CTs and two lung TTACs were determined, one from the high-density (HD) region of the IPF lung representing fibrosis and one from the low-density (LD) ‘normal appearing’ region. To achieve this, HD and LD masks (HDMask and LDMask) were created by initially segmenting the whole lung using a thresholding technique where the lung is considered any voxel with − 900 < HU < − 150 within the body boundary on the target CT. For the LDMask, the whole lung mask was eroded by three voxels (12 mm) to avoid edge effects. The ‘normal appearing’ tissue was assumed to occupy any voxel within this mask with HU < − 582. For the HDMask, the non-eroded whole mask is used, as the disease is most commonly seen in the lung periphery, and regions with HU > − 530 are considered fibrosis. The HDMasks were then manually edited (where necessary) to remove any areas in the ROI not representative of fibrosis.

Lung TTACs were determined by application of the HD and LD masks to the registered PET data. A two-tissue irreversible compartment model (Eq. 1) [20] was then fit to these lung TTACs using a weighted least squares method (Eq. 2).

1) (2 $$\begin{array}{@{}rcl@{}} C_{\mathrm{R}}(t) &=& V_{\mathrm{B}}C_{\mathrm{P}}(t) + K_{1}e^{-(k_{2}+k_{3})t}\otimes{C_{\mathrm{P}}(t)} + \frac{K_{1}k_{3}}{k_{2}+k_{3}}\left[1-e^{-(k_{2} + k_{3})t}\right]\otimes{C_{\mathrm{P}}(t)}\\ f(p)&=&\sum_{j=1}^{m}w_{j}(\psi_{j}(p)-y_{j})^{2} \end{array} $$

where *C*_R_ and *C*_P_ are the concentrations of the radiotracer in the region of interest and the plasma respectively; *K*_1_, *k*_2_ and *k*_3_ are the rate constants of the model; *V*_B_ is the fractional blood volume; ⊗ is the convolution operator; *j* indexes the time frames in the measured data; *ψ*_*j*_ is the model for each data point (computed as the numerical integral of Eq. 1 over time frame *j*), *p* are the parameters *p*=(*V*_B_,*K*_1_,*k*_2_,*k*_3_); *y*_*j*_ are the measured data; *f*(*p*) is the objective function for parameters *p*; *m* is the number of time frames; and *w*_*j*_ is a weighting factor equivalent to the inverse of the variance of the voxel values in the ROI making up the data point.

The parameters *K*_*i*2_ and *K*_*iP*_ [21] along with their standard errors were determined and compared. The standard error was calculated using the method outlined by Delforge et al. 1990 [30]. Briefly, the Hessian matrix (M) of *f*(*p*) is estimated using the Jacobian (J), the matrix of first order derivatives of the model *ψ*_*j*_ with respect to parameters *p*. Let W be a diagonal matrix with the *j*th diagonal element equal to the weights *w*_*j*_, the approximation of the Hessian can then be calculated:

3 $$ M=J'WJ  $$

with *J*^′^ the transpose of *J*. The parameter covariance matrix can then be determined:

4 $$ \text{COV}=\frac{f(p)}{m-r}M^{-1}  $$

where *r* is the number of parameters in the kinetic model. The estimated standard error, *s*_*k*_, on each parameter *p*_*k*_ is then:

5 $$ s_{k}=\sqrt{\text{COV}(kk)}  $$

This standard error is an estimate of the parameter error assuming that the model used is correct and that the only cause for errors is noise.

### Patient data: combined-CT method and analysis

The basic idea of the combined-CT method is to construct an AC map for each study section using all the available CT information. However, the process needs to take the changes in patient position into account.

**Fig. 1:**
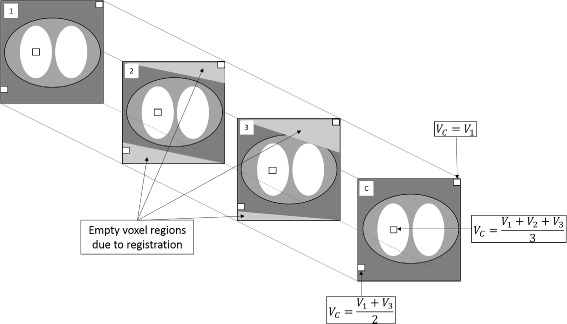
Schematic of the method to combine the CT data. 1, 2 and 3 represent the CT images from the early, mid and late study sections; C is the resulting combined-CT image; and V is the concentration in the voxel of interest. The white boxes represent specific voxels that are filled in either one, two or all of the CT images after registration. The grey areas at the edges of CT 2 and 3 are the result of registration

In a first step, the CTs from the different sections were registered to each other using the method previously described (the “Patient data: multi-PET/CT method and analysis” section). After registration, not all areas in the CT images will overlap due to variable positioning at the start of each study section (see Fig. 5) and because the late section contains a greater number of bed positions. Therefore, to create the combined-CT for each study section, the method shown schematically in Fig. 1 was used. This involved using a voxel-wise average across all voxel regions common to the CT for that section (the target CT) and the two registered CTs. Where the voxel was only present in two of the CT images, due to the registration, the average of the two voxel values was used to fill the appropriate voxel in the combined-CT image. Any remaining empty voxels were filled with the CT values from the target (unregistered) CT. This resulted in a new CT image where the entire field of view (FOV) was filled with a combination of the information from all the CT images.

The above process was repeated for each section. This enabled creation of a combined-CT in the space of each individual study section, avoiding possible issues that arise in registration due to mismatched field of view. The result is three combined-CT images, one in the space of each study section.

The raw PET data were then re-reconstructed and processed with the combined-CT images. TTACs were obtained using the HD and LDMasks defined in the “Patient data: multi-PET/CT method and analysis” section. The TTACs from the combined-CT and original-CT processing were visually compared, and *K*_*i*2_ and *K*_*iP*_ estimates and their associated standard errors were compared (the “Patient data: multi-PET/CT method and analysis” section). The density in each of the HD and LDMasks were also determined from each of the final combined-CTs after registration.

## Results

### XCAT simulations

**Fig. 2:**
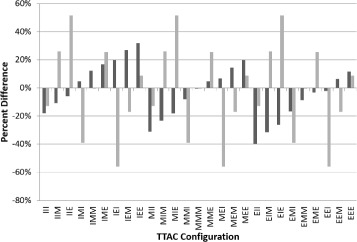
Variation in K _*i*_ due to respiration. The percentage difference between the true and measured K _*i*2_ (dark grey bars) and K _*iP*_ (light grey bars) in each of the different TTAC configurations where I, E and M represent ungated PET data reconstructed with an end inspiration, an end expiration and a mid-breathing cycle ‘snap shot’ CT for AC respectively (i.e. IEM = the early study section PET data were reconstructed with an end inspiration CT for AC, the mid section with an end expiration CT for AC and the late section with a mid-breathing cycle CT for AC)

Figure 2 displays the percentage differences between the true and the measured K _*i*_ for each of the different configurations of the TTACs. The error trend was found to be different for the compartment model and Patlak-Rutland methods of deriving the influx rate constant. As Patlak-Rutland only uses the final two study sections, the values of *K*_*iP*_ are not affected by the initial scan section, provided the IDIF is minimally affected by the early variations in density. The maximum errors in *K*_*iP*_ and *K*_*i*2_ were found to be 55.9 and 39.7% respectively.

### Patient analysis: multi-PET/CT

**Fig. 3:**
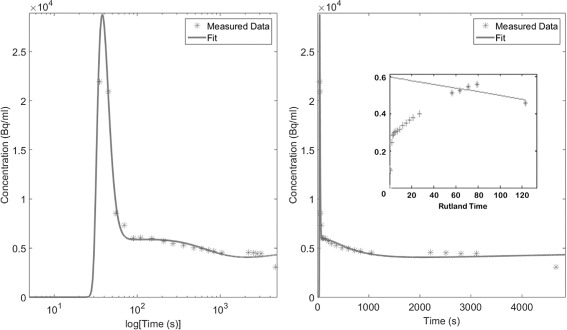
Example patient TTAC and fits with conventional analysis measured patient TTAC (stars) and the associated compartment model and Patlak-Rutland (inset to column 2) fits (filled line). Both columns show the same data. However, the left column has the *x*-axis in log(time) to allow better visualisation of the peak, while in the second column, the *x*-axis is linear in time

An example worst case (patient 5) FDG TTAC and the associated two-tissue compartment model and Patlak-Rutland fits are shown in Fig. 3. Discontinuity between the study sections is observed, which leads to a poor fit of the compartment model. The effect of these discontinuities also leads to incorrect analysis when applying Patlak-Rutland analysis. In this case, this resulted in a negative slope, which is inconsistent with the fact that FDG is considered an irreversible tracer over the time course of the study.

**Fig. 4:**
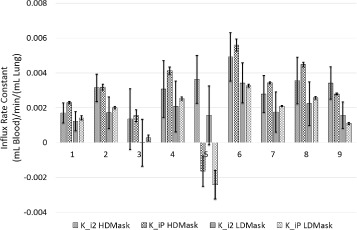
Regional variation in the K _*i*_ measurements with conventional analysis comparison of the fibrotic and normal appearing tissue influx rate constants using compartmental modelling (*K*_*i*2_) and Patlak-Rutland analysis (*K*_*iP*_) for all patients. Error bars represent standard errors

Figure 4 displays the measured influx rate constants from high- and low-density TTACs analysed with the two-tissue compartment model (*K*_*i*2_) and with Patlak-Rutland analysis (*K*_*iP*_) along with the associated standard errors. The percent error ((standard error /*K*_*i*_)×100*%*) for *K*_*i*2_ and *K*_*iP*_ was averaged for all patients and regions and found to be 44 ± 25% and 13 ± 17% respectively.

Theoretically, the two methods of calculating *K*_*i*_ (compartment model and Patlak-Rutland analysis) should render the same results. However, the maximum percentage difference between the estimated *K*_*i*_ parameters was found to be 250%. The average and standard deviation of the differences between the parameter estimates from the two analysis methods for the derived TTACs in the high and low density regions are 51 ± 102% and 43 ± 54% respectively.

### Patient analysis: combined-CT

**Fig. 5:**
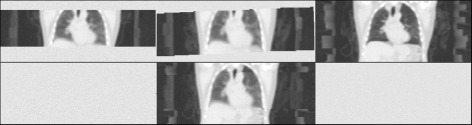
Example patient original and combined-CT images. The registered original CT images from the early (top left), mid (top middle) and late (top right) study sections and the resulting combined-CT for the late section (bottom middle) for an example patient. Grey scale − 1350 < HU < 150

Figure 5 displays the three registered original CT acquisitions for the example patient shown in Fig. 3 along with the combined late CT. On investigation of the CTs in this example patient, the lung density in the late acquisition was found to be up to 35% lower than that of the cine-CT acquisitions. Visual comparison of diaphragm position confirms that the CT was acquired at deep inspiration in the late stage of this study.

The density difference in the HD and LD lung between both the early and mid averaged cine-CTs and the late static CT are shown in Table 2. The maximum difference in lung densities between the early and mid averaged cine-CTs were found to be 10.0 and 12.5*%* respectively for the HD and LD regions. In contrast, the maximum difference in lung density between the early and mid combined-CT images were found be 1.4 and 1.2% in the HD and LD regions respectively.

**Table 2:** Average density variation in the HD and LD regions for all patients in the early and mid cine-CT studies with respect to the late ‘snap shot’ CT study (((*CT*_*x*_/*CT*_*L*_)−1)×100*%*) where *CT*_*x*_ is either *CT*_*E*_ or *CT*_*M*_)

	Original CT	Combined-CT
Early cine-CT HDMask	− 2.96 ± 9.09%	− 1.58 ± 4.96%
Early cine-CT LDMask	10.99 ± 16.66%	− 1.68 ± 4.84%
Mid cine-CT HDMask	2.80 ± 9.15%	1.06 ± 0.70%
Mid cine-CT LDMask	18.26 ± 18.27%	0.92 ± 0.32%

**Fig. 6:**
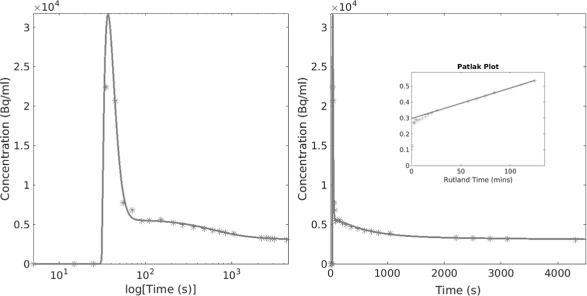
Example patient TTAC and fits with using combined-CT reconstructions for analysis. An FDG example TTAC with associated compartment model and Patlak-Rutland (inset) fits. This is the same FDG patient as shown in Fig. 3 for comparison. Both columns show the same data using different time axes as before (Fig. 3)

After reconstruction with the combined-CTs, all discontinuities in the PET-derived TTACs were reduced. Figure 6 displays the TTAC of the same example patient as shown in Fig. 3 with associated compartment model and Patlak-Rutland analysis fits. Analysis led to more consistent results when the combined-CT was used for reconstruction.

**Fig. 7:**
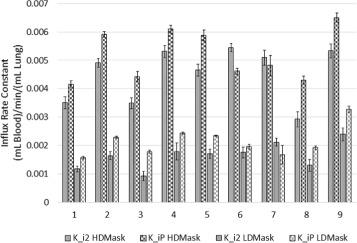
Regional variation in the K _*i*_ measurements using combined-CT reconstructions for analysis. Comparison of the fibrotic and normal appearing tissue influx rate constants determined after the PET data were reconstructed with the combined-CT, using compartmental modelling (*K*_*i*2_) and Patlak-Rutland analysis (*K*_*iP*_) for all patients. Error bars represent standard errors

Figure 7 displays the measured *K*_*i*2_ and *K*_*iP*_ from the high- and low-density TTACs along with the associated standard errors after the data have been reconstructed with the combined-CT for comparison with the original method shown in Fig. 4.

Table 3 displays the comparison between the estimates of *K*_*i*2_ and *K*_*iP*_. There is much better agreement using the combined-CT; a factor 2.5 and 1.7 for the high- and low-density regions respectively.

**Table 3:** Average ± standard deviation of the percentage differences between the compartment model and Patlak-Rutland analysis derived *K*_*i*_ estimates using the reconstructions with the actual acquired CTs (original CT) and using the reconstructions with the combined-CTs

	Original CT	Combined-CT
HDMask	51 ± 102%	20 ± 10%
LDMask	43 ± 54%	26 ± 11%

Table 4 displays the change in the standard errors for the original and new methods of reconstructing the PET data. For both *K*_*i*2_ and *K*_*iP*_ the data errors reduce to within 6%.

**Table 4:** The average parameter estimate errors as a fraction of the average parameter estimate for the FDG patients using the original acquired CTs and the combined-CTs for AC in the PET reconstruction at each study stage

	Original CT (%)	Combined-CT (%)
*K*_*i*2_ HDMask	53	6
*K*_*iP*_ HDMask	11	5
*K*_*i*2_ LDMask	50	5
*K*_*iP*_ LDMask	7	3

## Discussion

Quantitative imaging in the thorax is known to be complex due to respiratory effects. While motion correction has been extensively reviewed in the literature, little attention has been given to density changes. In previous work [15], we have shown, for the lung, that failure to account for CT density changes within the lung can lead to significant errors in estimated kinetic parameters, and in this paper, we have extended this work to investigate the effect of density variation in multi-PET/CT studies.

In simulations with ‘snap shot’ CTs, the largest errors in the parameter estimates are seen when the AC maps for each study section are acquired at different phases of the breathing cycle (Fig. 2). Accounting for CT density changes to ensure parameter stability is therefore essential for MPC studies including those that involve monitoring of disease progression over time.

With simulations appropriate for non-TOF ^18^F-FDG studies, it has been shown that MPC protocols can lead to errors in *K*_*i*_ of up to 57% with ‘snap shot’ CTs. The Patlak-Rutland influx rate constant is determined from the tail of the TTACs only and is largely unaffected by early study section errors. This was reflected in the fact that the Patlak-Rutland derived parameters were more precise and stable than the compartment model estimates. This was also found to be the case in the patient data (Table 4) where the standard errors were found to be 5 times greater for *K*_*i*2_ than *K*_*iP*_ before use of the combined-CT for AC.

The ^18^F-FDG protocol outlined in the “Patient acquisitions” section was split into three sections to encourage patient compliance. The derived TTACs from these data were found to have discontinuities between the study sections. The evidence suggests that this is associated with a variation in density across the CT acquisitions used for the AC at each stage. This variation in density could be due to timing of the short ‘snap-shot’ CT scans or, for averaged cine-CTs, possible variation in the breathing pattern between acquisitions.

For MPC studies, we propose that an approach for improving the match between the PET and CT in terms of density is to use an average of all the CT information to produce a combined-CT image (Fig. 5) for each study section. Results from applying the combined-CTs to each study section of the MPC protocols rectified TTAC discontinuity and improved parameter estimation (Fig. 6).

It can be observed from Figs. 4 and 7 that use of the combined-CTs produces absolute *K*_*i*_ values that are different to those obtained using the multiple CTs. This is due to the derived TTACs being very different and therefore producing very different parameter estimates. This is especially true as the model used to fit the TTAC from the original method could be considered to be wrong in this case.

Using the combined-CT approach, the average ratio of rate constants increased to 2.8 compared to 1.7 when using the original approach. This suggests that the proposed analysis may result in better discrimination of diseased and normal lung tissue, which would be important in evaluation of treatment.

Combining the CTs results in all the CTs used for AC having almost the same density in the regions where all the CTs overlap. However, it is noted that due to the larger FOV in the late section, there will be regions of lung that will not benefit from the combined-CT but nor can these regions be used for dynamic analysis.

Creating combined-CT images from ‘snap shot’ CT acquisitions has the advantage that a lower radiation dose will be given to the patient than use of cine-CTs. Good PET matching, however, is dependent on those ‘snap shot’ CTs being acquired at different stages of the breathing cycle, which cannot be guaranteed, unless instructed breathing is part of the clinical protocol, but patient compliance can be quite difficult to achieve, even with training [12]. Averaged cine-CTs, as available in the early and mid stages of this study, have the advantage that they more closely match the longer acquisition PET and would therefore be recommended as they represent entire respiratory cycles. However, in this work, the averaged cine-CTs from the early and mid study sections were found to still have variable densities in the lung. Moreover, the higher dose may mean that this method is not suitable for routine use.

The results in this paper show that the proposed approach of combining CTs is recommended for MPC studies involving ‘snap shot’ CTs, cine-CTs or a mixture of the two. However, it is noted that due to the irregularity of breathing, the combined-CTs may not be perfectly matched to the PET. The result is that the possibility of remaining mismatch artefacts and parameter bias still exists.

As an alternative to using the combined-CT method, our simulations indicate that training the patient such that a MID-CT can be acquired would work; however, as previously discussed, this may be difficult to achieve.

This paper has discussed mismatch between ungated PET and CT only. It should also be possible to gate the PET study and only reconstruct the gate that best matches the acquired CT. However, reconstructing gated dynamic PET data is challenging due to noise, especially in the early study frames. In addition, this approach would need to ensure that CTs in each section are acquired in the same respiratory state. Another alternative method is to use a joint registration/reconstruction technique that accounts for motion and misaligned attenuation, such as that outlined by Bousse et al. 2016 [31]. However, this technique would need adaptation to include corrections for the density changes for both the CT and gated PET data [32], but shows strong promise for the future of accurate quantitative pulmonary PET/CT imaging.

## Conclusions

Changes in respiratory induced lung density between PET and CT acquisitions contribute to the errors in lung tissue PET quantitation, especially in multi-PET/CT protocols, resulting in discontinuities between study sections in the time activity curves. When reconstructing the PET data for each study section, use of a combined-CTs for AC can improve the precision of the estimated parameters and may discriminate better between diseased and normal lung.
